# Neuropharmacological Assessment of the Hydroethanolic Leaf Extract of *Calotropis procera* (Ait). R. Br. (Apocynaceae) in Mice

**DOI:** 10.1155/2021/5551380

**Published:** 2021-07-01

**Authors:** Ernest Obese, Elvis Ofori Ameyaw, Robert Peter Biney, Emmanuel Awintiig Adakudugu, Eric Woode

**Affiliations:** ^1^School of Pharmacy and Pharmaceutical Sciences, College of Health & Allied Sciences, University of Cape Coast, Cape Coast, Ghana; ^2^Department of Pharmacology, School of Pharmacy, University of Health and Allied Sciences, Ho, Ghana

## Abstract

**Background:**

*Calotropis procera* has been widely used traditionally for its analgesic and anti-inflammatory effects. It is also reportedly used in ethnomedicine for mental health disorders including epilepsy even in the absence of supporting scientific data. Thus, the potential of the plant to affect neurological functions was evaluated.

**Methods:**

Irwin's test was performed to determine the effect of the oral administration of the extract (30–3000 mg kg^−1^) on gross behaviour and physiological function. The activity meter, rotarod, pentylenetetrazol- (PTZ-) induced convulsion, pentobarbitone-induced sleep test, and the tail immersion tests were used to evaluate the spontaneous activity, neuromuscular function, convulsive threshold, sedation, and analgesic effects of the *Calotropis procera* extract (30–1000 mg/kg), respectively, in mice.

**Results:**

*Calotropis procera* extract (CPE) exhibited significant (*p* < 0.0001) anticonvulsant and analgesic effects. There was a significant increase in withdrawal latency of the CPE-treated animals in the tail immersion test for analgesia (*p* < 0.0001), while latency and duration of PTZ-induced convulsions were positively modulated. *Calotropis procera* extract showed significant (*p* < 0.0001) central nervous system depressant effects in pentobarbitone-induced hypnosis at 100–1000 mg/kg and spontaneous activity test (30–1000 mg/kg). The extract also depicted impaired motor coordination at 100–1000 mg/kg dose levels. LD_50_ was estimated to be above 1000 mg kg^−1^.

**Conclusions:**

*Calotropis procera* extract has significant central nervous system depressant and analgesic effects in mice.

## 1. Background

Cognitive and neurological disorders are a major challenge for public health globally, particularly in developing countries where cultural factors and inadequate access to modern healthcare have led to dependence on conventional medicines [1]. These neurological disorders tend to be the leading cause of impairment worldwide, and their proportion among all health problems to the overall burden is growing. The brunt of the burden from neurological conditions lies in low- and middle-income countries [2].

In recent times, significant attempts have been made to identify drugs that may help prevent severe neurological disorders. Products of natural origin are small molecules that are present in various natural sources. They have a prestigious role in the treatment of all human diseases including neurological disorders [3]. It is thought that they are the single most powerful source of drug leads [4]. The relevance of plant-derived natural products for the treatment of neurological disorders is clear from the fact that most of the earlier medicines used to treat neurological disorders were derived from plants.

As part of the continuing search for plants with central nervous system activity, the potential of *Calotropis procera* (Ait) R. Br. (Apocynaceae) to affect CNS using mice was assessed. Despite the widespread use of the plant as an anti-inflammatory [5], anticancer [6], antimicrobial [7], and analgesic agent [8], very little scientific information exists about its effects on the CNS, although it is used traditionally in the management of epilepsy and some mental disorders [9]. There is, therefore, the need to evaluate this claim scientifically and provide support or otherwise for its ethnomedicinal uses in CNS disorders.

Until recently, the brain has been regarded as an immune-privileged organ, which was not susceptible to inflammation or immune activation and was thought to be largely unaffected by systemic inflammatory and immune responses. This view has been revised significantly with the realization that proinflammatory cytokines and other mediators play an essential role in CNS inflammation through microglia activation and its downstream mechanisms that lead to neurodegenerative effects. This mechanism is now recognized in several CNS disorders such as depression, Parkinson's disease, Alzheimer's disease, and epilepsy [10]. With the *C. procera* exhibiting potent anti-inflammatory property, it is hypothesized that it would possess some neuropharmacological effects in disorders, in which inflammation is known to play a role. Latex proteins of *C. procera* have been shown to have CNS activity as reflected in their potentiation of pentobarbital-induced sleeping time and their anticonvulsant action in a PTZ-induced seizure model. This demonstrates that *C. procera* has the ability to cross the blood-brain barrier [11].

The methods employed in this study were adapted from the core battery of assessment of the central nervous system as proposed by the International Conference on Harmonization (ICH) S7A Guideline for Safety Pharmacology [12]. It recommends the testing of novel compounds/extracts on the central and peripheral nervous system as part of the “core battery” of assessment for safety and efficacy [13]. These are generally simple tests employed in safety assessment and are frequently applied at the very beginning of the discovery process to screen substances with a potential for CNS benefit or risk and measure gross behavioural signs, locomotor activity, seizure, and pain thresholds. Irwin's test was employed to determine the effect of the oral administration of the extract on gross behaviour and physiological function. An activity meter cage was used to determine the effect of *C. procera* on locomotor activity, while the effects of the plant extract on neuromuscular coordination were assessed using the rotarod test. The analgesic activity of the plant was evaluated in a tail immersion assay. The sleep-enhancing effects and potential anticonvulsant actions were determined using a pentobarbitone-induced sleeping time and pentylenetetrazol-induced seizure models, respectively.

## 2. Materials and Methods

### 2.1. Plant Collection and Extract Preparation

Fresh leaves of *Calotropis procera* were collected from Iture (5°05′54.6″N, 1°18′48.7″W), a town near the University of Cape Coast (UCC), from August to December 2015. The leaves were identified by a botanist at the School of Biological Sciences Herbarium, the University of Cape Coast, and the voucher specimen (UCC/SBSH/15/M044) was deposited at the Herbarium. It was dried for fourteen days under shade and powdered using a hammer mill. The powdered sample was extracted as previously described [14]. An amount of 200 g of the powdered leaves was extracted with 2 L of 70% ethanol for 48 h using a Soxhlet apparatus (Aldrich® Soxhlet Extraction Apparatus, Z556203, St. Louis, MO, USA). The extract obtained was subsequently concentrated using a rotary evaporator (Rotavapor R-215, BÜCHI Labortechnik AG, Flawil, Switzerland) under reduced pressure and temperature (50°C). This was further dried to powder on a water bath, labeled as CPE (*Calotropis procera* extract), and then preserved in a desiccator containing activated silica until it was ready for use. The yield obtained was 4.8% *w*/*w*. The extract was reconstituted for use in the experiments by gently triturating to prepare a solution of it with distilled water as the vehicle.

### 2.2. Animals

Male ICR (Institute of Cancer Research) mice weighing 20–25 g were purchased from the Noguchi Memorial Institute for Medical Research (NMIMR), University of Ghana, Accra. They were kept in the animal house of the School of Biological Sciences, UCC, for seven days to acclimatize before the experiments. The animals were housed in cages (34 × 47 × 18 cm^3^) with softwood shavings as bedding and were maintained at a 12 h light-dark cycle. They had free access to food and water. The studies were conducted by NIH Guidelines for Care and Use of Laboratory Animals with approval from the Department of Pharmacology Ethics Committee.

### 2.3. Drugs and Chemicals

Morphine was purchased from Phyto-Riker, Accra, Ghana. Pentobarbitone, caffeine, *d*-tubocurarine, pentylenetetrazol, and diazepam were obtained from Sigma-Aldrich, St Louis, MO, USA.

### 2.4. Irwin's Test

The effects of *Calotropis procera* extract (CPE) on gross behaviour and physiological function were investigated using the original procedure described by Irwin [15]. Mice were randomly distributed to six groups (*n* = 7) and left to acclimatize for 24 h. Animals were fasted overnight but had free access to water. They were treated with oral doses of either CPE 30, 100, 300, 1000, 3000 mg kg^−1^, or distilled water 10 ml kg^−1^. The animals were subsequently observed for changes in behaviour and physiological function. Parameters such as analgesia, locomotor activity, response to touch, and death are scored using a rigorous standardized procedure based on the one described by Irwin at 0 to 15, 30, 60, 120, 180 min, and at 24 h.

### 2.5. Activity Meter Test

The effects of CPE on spontaneous locomotion [16] were evaluated with the Ugo Basile activity cage (model 7401, Comerio, VA, Italy). Mice were distributed randomly to seven groups (*n* = 7) and treated orally with either extract (30, 100, 300, or 1000 mg kg^−1^), diazepam (8 mg kg^−1^), caffeine (16 mg kg^−1^), or distilled water (10 mL/kg, p.o.). After 1 h, the animals were individually placed in the activity meter cage and their activities were scored in 5 min blocks for 30 min. Diazepam and caffeine were used as reference CNS depressant and stimulant, respectively. Total activity in 30 min was computed as the AUC of the time-course curve.

### 2.6. Rotarod Test

This test was performed to elucidate the effect of *Calotropis procera* extract on neuromuscular coordination. The rotarod consisted of a rotating rod (diameter: 3 cm) rotating at a constant speed of 25 revs/s with individual compartments for each mouse such that each mouse is physically separated from the other mice (Ugo Basile model 7600, Comerio, VA, Italy). Mice were trained for three days to stay on the rotating rod for 180 s. On the test day (24 h after the last training session), the animals received orally CPE (30, 100, 300, and 1000 mg kg^−1^), diazepam (8 mg kg^−1^), d-tubocurarine (0.01 mg kg^−1^), or distilled water (10 ml kg^−1^ p.o.) and placed on the rotating rod to walk. Latency to fall off the rotating rod within a maximum cut-off time of 180 s was determined at 0, 1, 1.5, 2 h, and 3 h after drug administration [17].

### 2.7. Pentobarbitone-Induced Sleeping Time

The effect of CPE on pentobarbitone-induced sleeping time was investigated in the pentobarbitone interaction test. The method described in [17] was adopted. Mice in seven groups (*n* = 7) received either CPE (30, 100, 300, and 1000 mg kg^−1^, *p.o.*), diazepam (8 mg kg^−1^), caffeine (16 mg kg^−1^), or distilled water (10 ml kg^−1^, *p.o.*) orally. Sodium pentobarbitone (50 mg kg^−1^) was administered intraperitoneally one hour after the respective drug treatments. Latency to sleep (time between pentobarbitone injection and loss of righting reflex) and duration of sleep (time between loss of and regaining of righting reflex) were recorded.

### 2.8. Convulsive Threshold Test

Mice were randomly assigned to five groups and administered either CPE (100, 300, and 1000 mg kg^−1^), diazepam (10 mg kg^−1^), or distilled water (10 ml kg^−1^, p.o.). One hour after drug treatment, the seizure was induced by a single subcutaneous dose of pentylenetetrazol (85 mg kg^−1^) at the nape of the neck. The mice were subsequently placed individually in plastic cages (Perspex chamber (15 × 15 × 15 cm)) for observation [17]. Latency to convulsion and frequency and duration of clonic and tonic convulsions were observed through video recording (Sony-Handycam, model: HDRCX675/B, Tokyo, Japan) for 30 min and quantified with the behavioural analysis software, JWatcher TM version 1.0 (University of California, Los Angeles, USA, and Macquarie University, Sydney, Australia; available at http://www.jwatcher.ucla.edu). Clonic seizures were characterized as the appearance of facial myoclonus, forepaw myoclonus, and forelimb clonus, and tonic seizures were characterized as explosive clonic seizures with wild running and tonic forelimb and hind limb extension. The latency for the onset, frequency, and duration of the convulsive episodes (clonic or tonic) was recorded as indicators of pro- or anticonvulsive effect of CPE.

### 2.9. Tail Immersion Test

The test was carried out according to the method described by [18] with some modifications. The distal part (2-3 cm) of the tail of the mice was immersed in a water bath maintained at 50.0 ± 1.0°C. The time in seconds to deflect or withdraw the tail out of the water was taken as the reaction time (T). A cut-off time of tail immersion was taken as 10 s, and thereafter the measurement was stopped to avoid any tissue injury. Withdrawal latency was taken at 0.5, 1, 2, and 3 h after the administration of CPE (30, 100, and 300 mg kg^−1^, p.o.) or morphine (10 mg kg^−1^, p.o.). Prior to the tail immersion test, the animals were screened by immersing their tail in hot water (50.0 ± 1.0°C) and only those animals which showed tail withdrawal latency of <5 s were selected for the experiment. An increase in tail withdrawal latency was the measure of antinociception which was calculated as

(1) $$\begin{array}{c} \%\text{ Maximal Possible Effect}\left(\text{MPE}\right)=\frac{\left[\left(T_{1}-T_{0}\right)\right]}{\left[\left(T_{2}-T_{0}\right)\right]}\times 100, \end{array}$$

where *T*_0_ and *T*_1_ are defined as the latencies obtained before and after drug treatment, respectively, and *T*_2_ is the cut-off latency.

### 2.10. Data Analysis

All results are presented as mean ± SEM. Data were analyzed using both one-way analysis of variance (ANOVA) and two-way ANOVA. When ANOVA was significant, multiple comparisons between treatments were performed using the Tukey *post hoc* test. GraphPad Prism for Windows, Version 7 (GraphPad Software, San Diego, USA), was used for all statistical analyses.

## 3. Results

### 3.1. Irwin's Test

The drug candidate, CPE (30–3000 mg kg^−1^), did not have any lethal effects on the animals during the 24-hour period of observation. The CPE-treated animals, however, showed analgesia, sedation, and decreased reactivity to touch at all doses administered compared to control. These effects lasted for 3 h after drug treatment. Also, no adverse reactions like convulsions, tremors, altered respiration, and death were observed.

### 3.2. Activity Meter Test

**Figure 1:**
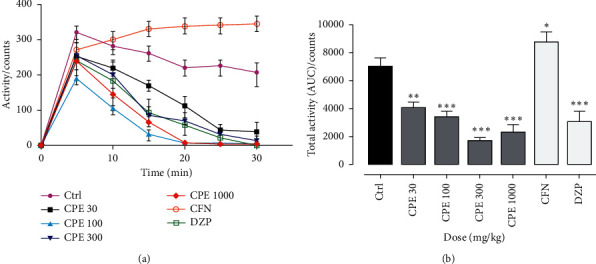
Time-course curve (a) and AUC of total activity (b) of mice administered CPE and diazepam in activity meter over a 30 min test period. Data are mean ± SEM, *n* = 7, ^*∗∗∗*^*P* < 0.0001, ^*∗∗*^*P* < 0.001, and ^*∗*^*P* < 0.05 compared to control (two-way ANOVA for the time-course curve followed by Tukey post hoc test and one-way ANOVA for the column graph followed by Tukey post hoc test).

The CPE significantly reduced spontaneous locomotion at doses of 30–1000 mg kg^−1^ as did diazepam 8 mg kg^−1^, the reference CNS depressant (*F*_6,42_ = 23.66 *P* < 0.0001). As shown in the time-course graph, the effect of the extract exhibited a significant reduction in locomotion compared to the control 10 min of test observation. This reduction was seen at all doses of the extract and continued to significantly decrease throughout the experiment. Caffeine, the reference CNS stimulant at 16 mg kg^−1^, increased activity significantly (Figure 1).

### 3.3. Rotarod Test

**Figure 2:**
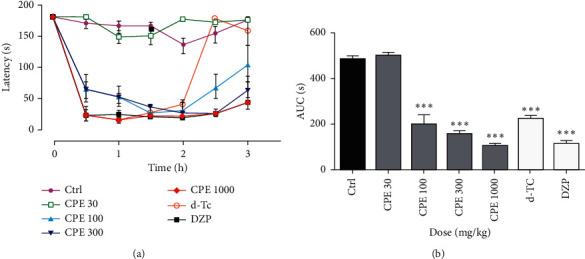
Effect of *Calotropis procera* extract (CPE), diazepam (DZP), and d-tubocurarine (d-Tc) on neuromuscular coordination in mice in the rotarod test. Time-course curve of duration spent on the rod (a) and total latency (AUC) (b) over 2 h data are presented as mean ± SEM, *n* = 7, ^*∗∗∗*^*P* < 0.0001 compared to control (two-way ANOVA for the time-course curve and one-way ANOVA for the column graph followed by Tukey's post hoc test).

The CPE reduced significantly the time spent on the rod only at doses of 100–1000 mg kg^−1^ (*F*_6, 39_ = 73.36, *P* < 0.0001). Lower doses of the extract could not alter the time spent on the rotating rod. The reference muscle relaxants, diazepam (8 mg kg^−1^) and d-tubocurarine (0.01 mg kg^−1^), also significantly reduced time spent on the rod (Figure 2).

### 3.4. Pentobarbitone-Induced Sleeping Time Test

**Figure 3:**
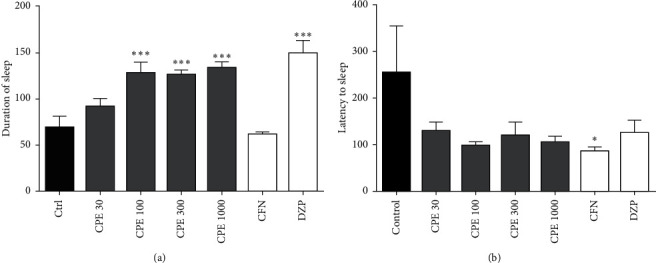
Effect of *Calotropis procera* extract on (a) duration of sleep and (b) latency to sleep in mice. Data are mean ± SEM, *n* = 7, ^*∗∗∗*^*P* < 0.0001, ^*∗∗*^*P* < 0.001, and ^*∗*^*P* < 0.05 compared to control (one-way ANOVA followed by Tukey's post *hoc* test).

The extract did not significantly affect the latency to sleep; however, it significantly prolonged sleep duration at doses of 100 to 1000 mg kg^−1^ (*F*_6, 41_ = 7.804, *P* < 0.0001). Diazepam, the reference CNS depressant, increased the duration of sleep while caffeine significantly decreased latency sleep duration (Figure 3).

### 3.5. Convulsive Threshold Test

**Figure 4:**
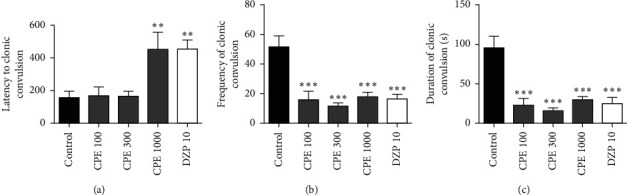
Effect of *Calotropis procera* extract on (a) latency, (b) frequency, and (c) duration of clonic convulsions in mice.

**Figure 5:**
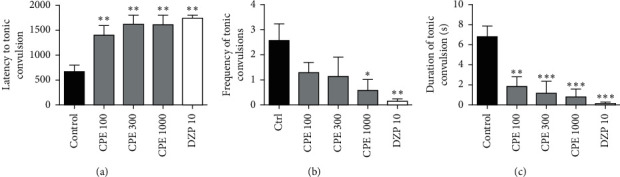
Effect of *Calotropis procera* extract on (a) latency, (b) frequency, and (c) duration of tonic convulsions in mice.

**Figure 6:**
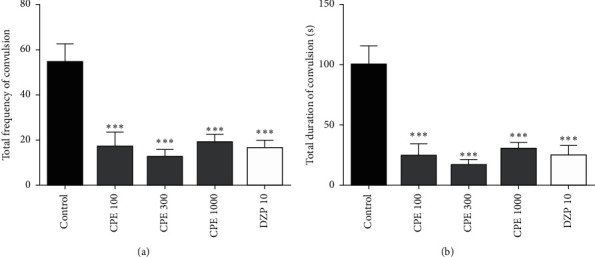
Effect of CPE on the (a) total frequency and (b) total duration of convulsions in mice. Data are mean ± SEM, *n* = 7, ^*∗∗∗*^*P* < 0.0001, ^*∗∗*^*P* < 0.001, and ^*∗*^*P* < 0.05 compared to control (one-way ANOVA followed by Tukey's post *hoc* test).

CPE (100–1000 mg kg^−1^) was able to significantly reduce the frequency (*F*_4, 30_ = 11.79, *P* < 0.0001) and duration (*F*_4, 30_ = 14.05, *P* < 0.0001) of clonic convulsions. However, only 1000 mg kg^−1^ of CPE was able to significantly increase the latency of the clonic convulsions (*F*_4, 30_ = 6.479, *P* < 0.0007). This is shown in Figure 4. The extract (100–1000 mg kg^−1^) also significantly reduced the duration (*F*_4, 27_ = 6.297, *P*=0.0010) and delayed the onset of tonic convulsions (*F*_4, 27_ = 4.882, *P*=0.0043). Lower doses of CPE were unable to reduce the frequency of tonic convulsions; however, the 1000 mg kg^−1^ dose of the extract significantly reduced the frequency of tonic convulsions (*F*_4, 27_ = 2.997, *P*=0.0247) (Figure 5). Overall, CPE was able to reduce the total frequency (*F*_4, 30_ = 10.33, *P* < 0.0001), and duration (*F*_4, 30_ = 12.93, *P* < 0.0001) of convulsions significantly (Figure 6). The reference anticonvulsant used (diazepam) was also able to reduce the frequency and duration of the convulsions and delay the onset of tonic convulsions.

### 3.6. Tail Immersion Test

**Figure 7:**
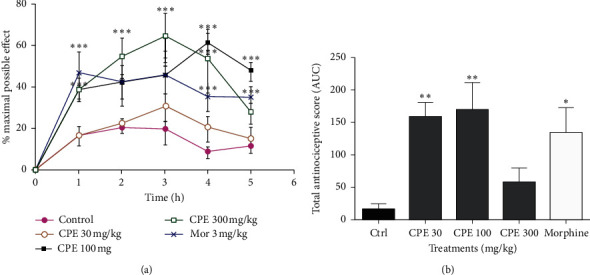
Effect of CPE (30–300 mg kg^−1^, *p.o*.) and morphine (10 mg kg^−1^, i.p.) on the time-course curve (a) of the tail immersion test and the AUC (b) in mice. Data are presented as mean ± S.E.M. ^*∗*^*P* < 0.05, ^*∗∗*^*P* < 0.001, and ^*∗∗∗*^*P* < 0.0001 compared to the control group (two-way ANOVA followed by Tukey's *post hoc* test for the time-course curve or one-way ANOVA followed by Tukey's *post hoc* test for AUC).

From the time-course curves in Figure 7, two-way ANOVA (*treatment* × *time*) revealed a significant effect of drug treatments on the tail withdrawal latencies calculated as a percentage of the maximum possible effect (% MPE) (*F*_5,15_ = 55.06, *P* < 0.0001). CPE (30–100 mg kg^−1^, *p.o*.) significantly increased tail withdrawal latency (*F*_4,14_ = 5.440, *P*=0.0074). Morphine (10 mg kg^−1^, i.p.), the standard analgesic drug used, also showed a significant increase in the withdrawal latency (*P* < 0.05).

## 4. Discussion

This present study has shown that the hydroethanolic leaf extract of *Calotropis procera* possesses CNS depressant, anticonvulsant, analgesic, and muscle relaxant effects in the animal models used.

The mice treated with CPE showed signs of sedation and analgesia, suggesting possible central depressant and analgesic effects in Irwin's test. This quite simple test can provide pertinent information about a potential therapeutic indication, a specific mechanism of action, or a specific physiological function. For example, the presence of sedation in this test could be suggestive of a possible anxiolytic, antipsychotic, or anticonvulsant activity [19]. Irwin's test also assesses the minimum lethal dose of a test substance and the primary effects on behaviour and physiological functions. Data from this test can also be used to assess the safety pharmacology of investigational drug agents [15]. In the first 24 h after administration, the plant extract caused no mortality and appeared to cause no apparent toxicity even at a relatively high dose. This result suggests that orally administered CPE is relatively nontoxic, since substances with an estimated LD_50_ tend to be above 1000 mg kg^−1^. It can thus be regarded as being safe or of low toxicity [20].

Locomotor activity is used to assess whether there is a psychostimulant or sedative activity in a new drug substance. The distinction between Irwin's estimates of the drug effects on random activity tests and activity meter tests is the quantification process [16]. Locomotor activity is needed for many complex behavioural tasks, and, in many behavioural tests, increases or decreases in locomotor activity affect performance in such tasks [21]. One of the most common side effects of commonly used sedatives is the risk of impairing psychomotor functions [22]. Usually, central nervous system activation leads to increased activity, whereas CNS depression reduces activity levels [23, 24]. Thus, the activity meter test sought to confirm some observations such as sedation and the reduced touch responses as observed in Irwin's test and assessed the effects of *Calotropis procera* extract on spontaneous locomotion. It was observed from the experiment that the activity of the test animals was significantly reduced in all the doses of CPE tested (30–1000 mg/kg). This reduced locomotion may be due to sedation, drug-induced motor weakness, or debilitation by the test agent [25].

The rotarod test was conducted to determine whether the decrease in spontaneous movement was due to drug-induced motor dysfunction or sedation. Mice output on a rotarod is a responsive and commonly used tool for evaluating motor functional aspects of balance and coordination [26, 27]; therefore, muscle synchronization and balance in the fore and hind limbs may be studied. This role demands an intact cerebellar and motor coordination function [28, 29]. Mice with serious issues with motor control would have trouble remaining on the spinning rod. From the results, it was clear that there was significant motor impairment at the doses of the extract that reduced spontaneous activity in the activity meter test. Many CNS depressant compounds can cause a reduction in spontaneous locomotor activity in laboratory animals. Nearly all the neuroleptic agents used in psychiatry diminish spontaneous locomotor activity in all species including man [30, 31]. It was observed in the experiment that diazepam, a CNS depressant, reduced spontaneous locomotive activity and impaired motor coordination at the doses used, while caffeine, a CNS stimulant, increased the locomotor activity [32, 33].

The ability of substances to cause sleep-enhancing effects can readily be detected in the barbiturate-induced sleeping time test by substances, which do not cause sleep even at high doses when given alone [34, 35]. It has been found that the results found in this experiment are strongly associated with those observed in other certain complex experiments and in humans [36]. Barbiturates are reputed sedatives that induce human and animal sleep by causing depressing of the central nervous system [37]. Pentobarbitone potentiates the effect of GABA, acting at the GABA receptor-ionophore complex [38]. The activation of GABA_A_ receptors depresses the CNS and thus favours sleep; therefore, an increase or a decrease in the pentobarbitone-induced sleeping time may be a useful method for investigating GABAergic system influences [39]. In the present study, though the extract did not significantly affect the latency to sleep, it profoundly prolonged sleep duration, and this was consistent with the observed sedation in Irwin's test. The potentiation of the pentobarbitone-induced sleep further supports the central depressant activity of the extract [40].

The pentylenetetrazol test is the most widely used acute chemical experimental model to identify new antiepileptic drugs [41]. One of the generally accepted mechanisms by which pentylenetetrazol exerts its action is by acting as an antagonist at the GABA_A_ receptor complex [42]. PTZ prevents GABA-mediated Cl^−^ influx in the Cl^−^ channel via an allosteric interaction, leading to convulsions in animals [43, 44]. For a long time, the GABAergic mechanism has been involved in epilepsy. Improving and inhibiting GABA's neurotransmission can attenuate and increase seizures, respectively [45, 46]. Deficiencies in GABA neurotransmission in both experimental animal models and human syndromes are associated with epilepsy [41, 47]. An agent's ability to avoid or postpone the onset of PTZ-induced clonic and tonic-clonic convulsions in animals is an indicator of anticonvulsant activity [48, 49]. The *Calotropis procera* extract produced significant inhibition of PTZ-induced seizures, which helps to confirm its traditional use in epilepsy management [9] as this is a well-validated test for anticonvulsants. It was observed that, in doses above 30 mg kg^−1^, the extract delayed the onset of clonic and tonic convulsions and also reduced the frequency and duration of the clonic and tonic convulsions. The potent effect of diazepam as evident in the PTZ-induced convulsions agrees with its enhancing effects in GABAergic neurotransmission [50]. GABA is a major inhibitory neurotransmitter in the central nervous system in humans [42]. Inhibition of pentylenetetrazol-induced seizures could indicate that the anticonvulsant effects of CPE may be linked with GABA activity modulation in the central nervous system. This may not be surprising as it has demonstrated significant central depressant properties.

During Irwin's test, it was seen that CPE (30–3000 mg/kg) produced analgesia. This observation was further confirmed in the tail immersion test, an acute thermal pain model [51], where morphine was the reference analgesic. In the tail immersion test, CPE caused a prolonged latency period, indicating an increase in the nociceptive threshold. The response to the tail-immersion test is a spinal reflex with the involvement of higher neural structures and is used to evaluate central analgesic activity [52]. There is, therefore, a possibility that the analgesic effect of the extract may be associated with spinal or supraspinal pathways. The antinociceptive effect of CPE in this test is a further confirmation of analgesia observed in Irwin's test.

The occurrence of several biologically active phytochemicals in various plant extracts such as flavonoids, triterpenes, alkaloids, steroids, tannins, and glycosides can be responsible for their respective pharmacological properties [53–55]. Screening of the plant extract in previous studies revealed the presence of tannins, saponins, terpenoids, flavonoids, and alkaloids [5]. The observed pharmacological activities of the extract in the various animal models used could be attributed to the presence of these phytochemicals. Previous research has shown that plants containing flavonoids, saponins, and tannins are beneficial in many CNS disorders [56, 57].

## 5. Conclusion

From the core CNS battery tests of the ICH S7A guidelines employed in this study, *Calotropis procera* extract has significant CNS depressant, anticonvulsant, and analgesic effects and is deemed to be relatively safe when administered orally.

## Data Availability

The datasets used and/or analyzed during the current study are available from the corresponding author upon request.
